# Engagement in Music-Related Activities During the COVID-19 Pandemic as a Mirror of Individual Differences in Musical Reward and Coping Strategies

**DOI:** 10.3389/fpsyg.2021.673772

**Published:** 2021-06-28

**Authors:** Laura Ferreri, Neomi Singer, Michael McPhee, Pablo Ripollés, Robert J. Zatorre, Ernest Mas-Herrero

**Affiliations:** ^1^Laboratoire d'Étude des Mécanismes Cognitifs, Université Lumière Lyon 2, Lyon, France; ^2^Montreal Neurological Institute, McGill University, Montreal, QC, Canada; ^3^Department of Psychology, New York University, New York, NY, United States; ^4^Music and Auditory Research Laboratory (MARL), New York University, New York, NY, United States; ^5^Center for Language, Music and Emotion (CLaME), New York University, New York, NY, United States; ^6^International Laboratory for Brain, Music and Sound Research, Montreal, QC, Canada; ^7^Department of Cognition, Development and Education Psychology, Universitat de Barcelona, Barcelona, Spain

**Keywords:** music, COVID-19, music reward, emotional regulation, musical abilities, individual differences

## Abstract

The COVID-19 pandemic and the measures taken to mitigate its impact (e.g., confinement orders) have affected people's lives in profound ways that would have been unimagable only months before the pandemic began. Media reports from the height of the pandemic's initial international surge frequently highlighted that many people were engaging in music-related activities (from singing and dancing to playing music from balconies and attending virtual concerts) to help them cope with the strain of the pandemic. Our first goal in this study was to investigate changes in music-related habits due to the pandemic. We also investigated whether engagement in distinct music-related activities (singing, listening, dancing, etc.) was associated with individual differences in musical reward, music perception, musical training, or emotional regulation strategies. To do so, we collected detailed (~1 h-long) surveys during the initial peak of shelter-in-place order implementation (May–June 2020) from over a thousand individuals across different Countries in which the pandemic was especially devastating at that time: the USA, Spain, and Italy. Our findings indicate that, on average, people spent more time in music-related activities while under confinement than they had before the pandemic. Notably, this change in behavior was dependent on individual differences in music reward sensitivity, and in emotional regulation strategies. Finally, the type of musical activity with which individuals engaged was further associated with the degree to which they used music as a way to regulate stress, to address the lack of social interaction (especially the individuals more concerned about the risk of contracting the virus), or to cheer themselves up (especially those who were more worried about the pandemic consequences). Identifying which music-related activities have been particularly sought for by the population as a means for coping with such heightened uncertainty and stress, and understanding the individual differences that underlie said propensities are crucial to implementing personalized music-based interventions that aim to reduce stress, anxiety, and depression symptoms.

## Introduction

“*What the pandemic has crystallized in my mind is that we need music because it helps us to get to very specific states of mind*,” the famous cellist Yo-Yo Ma recently declared^1^ The COVID-19 pandemic and the measures taken to slow the spread of the virus, in particular confinement orders, have profoundly changed the ways that people live, with profound implications for mental health, including widely documented increases in the prevalence of anxiety, depression, and stress (Mazza et al., 2020; see Xiong et al., 2020 for a review). Mitigating such hazardous effects of COVID-19 on mental health should therefore constitute an international public health priority (Xiong et al., 2020).

During March–April 2020, as shelter-in-place orders went into effect in many countries, media reports showed people from Italy and Spain singing and playing music on their balconies as a means of boosting morale during the lockdown. Through its ability to evoke strong emotions, music can profoundly alter listeners' internal states (Sloboda et al., 2001). Consistent research has shown that people mainly listen to music in order to modulate their emotional state (Juslin and Vastfjall, 2008; Lonsdale and North, 2011). For example, listening to music from childhood and youth effectively evokes intense emotional responses by specifically promoting autobiographical memories (Schulkind et al., 1999; Janata et al., 2007; Janata, 2009). Furthermore, listening to self-selected familiar music reduce loneliness and repair mood through comforting mechanisms (Schäfer and Eerola, 2020; Schäfer et al., 2020). Also, playing music or singing have been extensively shown to promote psychological well-being (Clift et al., 2010; Koelsch, 2013; D'Ausilio et al., 2015). As several types of musical behaviors can evoke a variety of emotions, people may engage in musical activities to address a variety of regulatory needs, such as achieving or maintaining a positive affective state, reducing or diverting attention from negative affective states, or finding solace in music (Juslin and Vastfjall, 2008; Rentfrow, 2012; Saarikallio, 2012). In this vein, one of the most notable attributes of music, which can be highlighted as a significant resource for mood regulation, is its ability to induce intensely pleasurable feelings (Zatorre and Salimpoor, 2013).

In a recent study, we showed that music was pivotal for coping with the negative consequences of the pandemic (Mas-Herrero et al., 2020). Among many daily and rewarding activities (i.e., physical exercise, food-related activities, drugs, and sex), musical activities such as music listening or singing emerged as the most effective for coping with the crisis and its associated psychological distress. Strikingly, we found that the relationship between increased engagement in musical activities and decreased depression symptoms was mediated by participants' sensitivity to reward (i.e., level of general hedonia, as measured by the Physical Anhedonia Scale, Chapman et al., 1976). Supported by a broad literature on music's benefits for general well-being (see e.g., Laukka, 2007; Gick, 2011; MacDonald et al., 2013; Osman et al., 2016), these important findings highlighted that (1) music is a useful tool for facing negative affective states related to the COVID-19 crisis and that (2) the beneficial effect of music on well-being is mainly driven by music's ability to induce pleasure.

Notably, musical pleasure is a complex, multifactorial construct that can vary according to specific cultural and personal preferences (Mas-Herrero et al., 2013). Indeed, music may lead to pleasurable reactions via different sources and activities, from passively listening to music to singing, dancing, or by providing a context of social interaction (see e.g., Lonsdale and North, 2009; Mas-Herrero et al., 2013; Witek et al., 2014). Importantly, large differences are observed across individuals in how musical pleasure is experienced (Mas-Herrero et al., 2013; Martínez-Molina et al., 2016). While some individuals may particularly enjoy listening to highly emotional excerpts, others may be specifically sensitive to dancing or music's ability to regulate their mood. Interindividual differences in these various facets of musical reward can provide important insights in determining the activities that drive the most pleasure for each individual and, therefore, personalize the optimal strategy to mitigate psychological distress (e.g., depression).

It is increasingly acknowledged that the idiosyncratic manner by which people regulate their emotions plays an important role in determining musical benefits on mental health (Chin and Rickard, 2014). Two commonly used emotion regulation strategies are cognitive reappraisal and expressive suppression (Gross and John, 2003). Cognitive reappraisal is considered an antecedent-focused process whereby an individual alters the emotional impact of a given situation by changing the way he thinks about it. Expressive suppression, on the other hand, is a response-focus process whereby an individual attempts to reduce the behavioral manifestation associated with an emotional response that is already in motion. Research indicates that cognitive reappraisal is more effective in reducing negative affect than expressive suppression (John and Gross, 2004). The consistent use of cognitive reappraisal correlates with healthy patterns of affect and with increased well-being, as well as with ameliorated depression or anxiety (John and Gross, 2004; Moore et al., 2008; Cutuli, 2014), including within the context of music-use for emotion regulation (Västfjäll et al., 2012; Chin and Rickard, 2014). In this regard, music-related activities promoting cognitive reappraisal regulation strategies may be particularly beneficial to mitigate mood-related disorders.

Although our previous work shows that musical activities resulted among the most effective to cope with the psychological distress induced by the COVID-19 pandemic (Mas-Herrero et al., 2020; see also Carlson et al., 2021; Fink et al., 2021; Gibbs and Egermann, 2021; Martín et al., 2021; Martínez-Castilla et al., 2021), the question arises as to how music-related habits changed during this period of crisis, and whether these changes in musical habits were associated with individual differences in musical and psychological (i.e., emotional regulation) profiles. The current study is based on the same sample and questionnaire described in Mas-Herrero et al. (2020). While the previous study (Mas-Herrero et al., 2020) aimed at comparing music to other rewarding daily activities (including exercise, cooking, reading, meditation, sex, drugs, etc.) during COVID-19 restriction, in the current study, we specifically focused on musical behaviors. In particular, here we aimed at (i) investigating changes in music-related habits as a result of the pandemic; (ii) determining whether engagement in distinct music-related activities was associated with individual differences in different facets of individuals' sensitivity to music reward (i.e., musical hedonia, Mas-Herrero et al., 2013), emotional regulation strategies, or musical training; and (iii) exploring which type of psychological support (i.e., to regulate stress, to address the lack of social interaction, or to cheer themselves up) people sought in music to mitigate their worries.

## Materials and Methods

### Participants

One thousand and thirty-one participants completed the survey. Participants were recruited via two modalities: (i) as unpaid volunteers using online postings, social media, and mailing lists; or (ii) as paid participants recruited from Amazon Mechanical Turk (AMT; a total of 477 were recruited in this way), which further ensured that no specific restrictions in terms of interests or psychological factors influenced participant's recruitment. All protocols were approved by the local institutional review board (New York University's Committee on Activities Involving Human Subjects). Only participants aged ≥18 years were included in the study. Fifty participants were discarded from the original sample for not completing several attentional checks placed within the survey. The final sample (the same as in Mas-Herrero et al., 2020) was composed of 981 participants distributed across Italy (*N* = 286), Spain (*N* = 295), the United States (*N* = 306), South America (*N* = 43), the rest of Europe (*N* = 26), and the rest of the World (*N* = 25). Importantly, the recruitment was not biased toward music (see Mas-Herrero et al., 2020, also for distribution of the demographics and socioeconomic status), and participants did not know that the study was focused on music until after they started answering the questions.

### Procedure (Online Survey)

Volunteers worldwide participated in the survey (see Supplementary Information) via Amazon Mechanical Turk or following a hyperlink. All participants approved the informed consent before starting the survey. Participants first provided demographic data and information about how the COVID-19 crisis affected them. They also indicated how worried they were about contracting the virus themselves, about their relatives and friends contracting the new coronavirus, and about the potential consequences of the pandemic in the future (from 0 = Not at all worried to 4 = Extremely worried). Then, they provided a subjective evaluation of whether they were engaging more or less in 43 different activities during the lockdown using a 1–7 Likert scale (1-Much Less, 7-Much More; 4-The same amount). Among these activities, only those involving music were analyzed in the current study (10 items). Concretely, five items were associated with *music listening* habits (listening to music in general, listening to sad music, listening to happy music, listening to music from childhood, and discovering and listening to new music). Four items were associated with *music production* (singing, dancing, playing an instrument, and composing/creating music). Finally, one item was about attending/watching virtual or recorded concerts. Next, participants were also asked to indicate how music (and other activities) helped them cope with the new COVID-19 emergency. Particularly, we asked them whether (1) music helped them to deal with the stress and worries generated by the COVID-19 crisis (“negative emotion regulation”: e.g., music helped me to deal with the current situation; music helped me to get rid of my worries; or music made me feel I have certain control over the situation), (2) music helped them to overcome the lack of social interaction (“socialization”: e.g., music kept me company, music made me feel less lonely or isolated, or music made me feel connected with others), or (3) music made them feel good (“positive feelings”: e.g., music comforted me; music helped me to perk up; music made me feel good). For each item, participants had to indicate how much they agreed or disagreed using a 1–7 Likert scale (from 1 = completely disagree to 7 = completely agree). Finally, participants also completed several questionnaires related to individual differences in emotion regulation strategies (Gross and John, 2003; Cabello et al., 2013; Emotion Regulation Questionnaire, ERQ; Balzarotti, 2019) and music reward sensitivity (Barcelona Music Reward Questionnaire, BMRQ, Mas-Herrero et al., 2013). ERQ includes 10 items (answers based on a 7-point Likert-type scale, from “strongly disagree” to “strongly agree”) assessing cognitive reappraisal and emotional suppression strategies. BMRQ measures individual sensitivity to music reward (i.e., musical hedonia) through a total 20 items (answers based on a 5-point Likert-type scale, from “strongly disagree” to “strongly agree”) assessing five subscales: music-related emotional evocation, mood regulation, sensori-motor responses, seeking behavior, and social reward. Musicianship was assessed through the musical training scale from the Goldsmith Musical Sophistication Index (Gold-MSI, Müllensiefen et al., 2014). Other questionnaires such as the Physical Anhedonia Scale and the Depression, Anxiety, and Stress Scale (DASS-21) were also administrated but reported in a previous manuscript (Mas-Herrero et al., 2020) and not considered in the current manuscript. The median total time for completing the survey was 39 min. All questions and questionnaires were translated into each of the three language spoken by participants (i.e., English, Spanish, and Italian). Validated translations of the questionnaires were employed when available. When missing, two native speakers worked together on a translation from English to Italian or Spanish; translated items were then evaluated by at least two independent reviewers (with Spanish or Italian as mother tongue), and further harmonized by the translators when necessary. In order to pre-test the correct functioning of the online testing and the clarity of the items, each version of the survey (i.e., in the three languages) was entirely tested by naïve participants before starting the data collection. At the time of the survey (May–June 2020), the main three countries tested (Italy, Spain, and United States) were at the end of the first lockdown restrictions (ended on May 18th in Italy, on May 10th in Spain, and between April 13th April and June 13th 2020 in the United States). Importantly, throughout the whole survey, participants were specifically asked to provide answers concerning the lockdown period, which for most individuals had only very recently ended.

### Analysis

Wilcoxon-Rank tests were used to assess whether individuals sought more or less music-related activities during the pandemic than before. To investigate the relationship between music habits during the lockdown and individual differences (i.e., in musical pleasure sensitivity, musical training, and emotional regulation strategies), we performed a linear mixed model for each item/habits (ten in total: listening to music in general, listening to sad music, listening to happy music, listening to music from childhood, discovering and listening to new music, singing, dancing, playing an instrument, composing/creating music, and attending/watching virtual or recorded concerts) with R (version 4.0.2) and RStudio (version 1.3.959) using the lme4 package. All models included random intercepts for each region (Spain, Italy, the USA, South America, the rest of Europe, and the rest of the World). All analyses included as fixed factors: the five facets of the BMRQ (Musical Seeking, Social Reward, Emotion Evocation, Emotion Regulation, and Sensory-Motor), the musical training score of the Gold-MSI, the two facets of the ERQ (Cognitive Reappraisal and Emotional Suppression), and potential confounding factors such as age, gender, and education level. *P*-values for the fixed factors were Bonferroni-corrected for multiple comparisons for the 10 models tested (α = 0.05/10).

To investigate the emotional regulatory reasons associated with each music-related activity, we averaged participants' responses on the items assessing whether music-related activities, in general, helped them to (1) handle the current situation (i.e., “negative emotion regulation” scale), (2) reduce the feeling of social isolation (i.e., “socialization” scale), and (3) to make them feel good, relaxed, etc. (i.e., “positive feelings” scale). Each scale was represented by five items. The three scales were chosen based on a large body of literature showing that affect regulation based on reappraisal, emotional social support, and reward are frequently reported in response to emotional episodes (Larsen, 2000; Livingstone and Srivastava, 2012; Páez et al., 2013), and are generally sought by individuals engaging in music-related activities to cope with negative emotional situations (Rentfrow, 2012; Saarikallio, 2012). To look at the relationship between participants' scores in these three scales and their engagement in different music-related activities, we ran independent linear mixed models for each scale. All models included participants' engagement in each of the 10 music-related activities and potential confounding factors such as age, gender, and education level as fixed-factors. In addition, and given the strong correlation between the three scales (*r*s > 0.69), on each model, the other two scales were also included as fixed factors (e.g., the model to predict the “socialization” scale includes the “negative emotion regulation” and “positive feelings” scales as fixed factors) to specifically test the effects of each music-related activity on each scale beyond the common effect of the three. Despite the high correlation between scales, multicollinearity did not present a problem in any models [all variance inflation factor (VIF) values <3]. As in the previous analysis, all models included random intercepts for each region. *P*-values for the fixed factors were Bonferroni-corrected for multiple comparisons (α = 0.05/3).

Finally, we explored the relationship between participants' responses in these three scales (i.e., negative emotion regulation, socialization, and positive feelings) and their worries about (i) the potential implication of the COVID-19 emergency and (ii) the possibility of contracting themselves or their loved ones the COVID-19. To do so, we ran a mixed-effect model for each concern, including the three previously described scales (negative emotion regulation, socialization, positive feelings) and potential confounders (age, gender, education level) as fixed factors. Both models included random intercepts for each world region. *P*-values for the fixed factors were Bonferroni-corrected for multiple comparisons (α = 0.05/2).

The different predictors' effects were assessed using the *car* package in R with Type II Wald chi-square tests.

## Results

**Figure 1:**
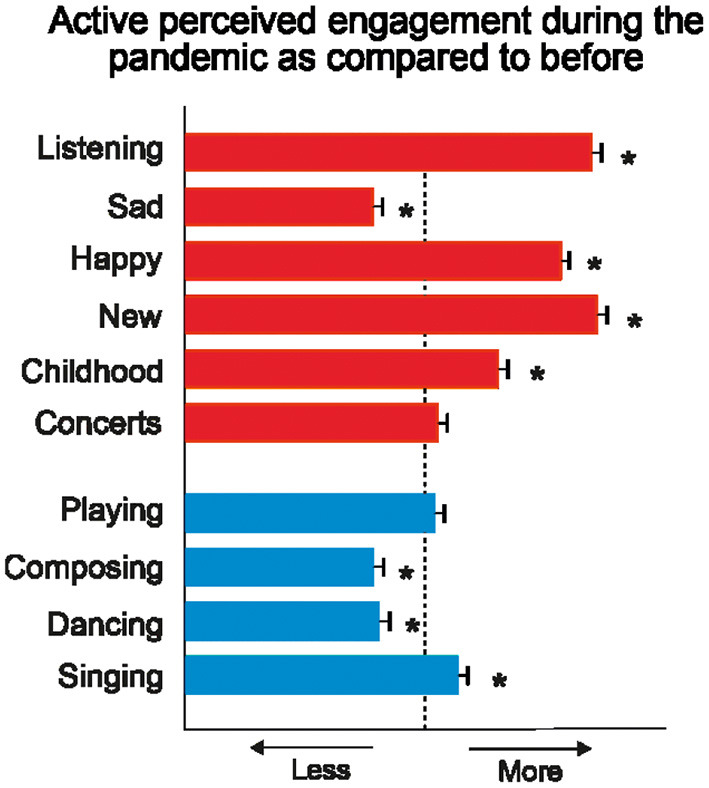
Subjective perceived changes of engagement in music-related activities involving music listening (in red; i.e., overall listening to music, listening to sad music, happy music, new music, music from their childhood, attending to virtual concerts) or production (in blue; i.e., music playing, composing, dancing, singing) during the pandemic as compared to before. The dash line represents no change in engagement. *p < 0.05.

First, we examined how much participants pursued music-related activities during the pandemic as compared to before (Figure 1). Concerning music listening habits, participants reported listening more to music (*Z* = 13.42, *p* < 0.001, *d* = 0.43), particularly to happy music (*Z* = 13.00, *p* < 0.001, *d* = 0.42), and both new music (*Z* = 13.87, *p* < 0.001, *d* = 0.44) and music from their childhood (*Z* = 6.44, *p* < 0.001, *d* = 0.21). On the contrary, sad music was less sought after during the COVID-19 emergency than before (*Z* = 5.50, *p* < 0.001, *d* = 0.18). No differences in attendance to virtual concerts or watching recorded concerts were found (*Z* = 0.70, *p* = 0.48, *d* = 0.02).

Regarding music production, participants reported singing more during the lockdown than before (*Z* = 2.64, *p* =0.008, *d* = 0.08). Yet, they spent less time dancing (*Z* = −4.71, *p* < 0.001, *d* = 0.15) and composing/creating music (*Z* = 5.70, *p* < 0.001, *d* = 0.18). No change in playing instruments was found significant (*Z* = 0.09, *p* = 0.93, *d* = 0.00).

We further aimed to assess the extent to which individuals' musical habits during the pandemic were associated with individual differences in music reward (using the five facets of the BMRQ: Musical Seeking, Emotion Evocation, Emotion Regulation, Sensory-Motor, Social Reward), musical training (through the GSI), and individuals differences in emotion regulation strategies (as measured by the ERQ; see Table 1).

**Table 1:** Chi-square and *p*-value (uncorrected) for the main predictors included in each model.

	**Listening**		**Sad**		**Happy**		**New**		**Childhood**		**Concerts**		**Playing**		**Composing**		**Dancing**		**Singing**	
	**χ^2^**	***p***	**χ^2^**	***p***	**χ^2^**	***p***	**χ^2^**	***p***	**χ^2^**	***p***	**χ^2^**	***p***	**χ^2^**	***p***	**χ^2^**	***p***	**χ^2^**	***p***	**χ^2^**	***p***
Intercept	5.76	0.016	**61.62**	**0.000**	**19.56**	**0.000**	**16.76**	**0.000**	**25.36**	**0.000**	**44.40**	**0.000**	**12.13**	**0.000**	**39.22**	**0.000**	**31.65**	**0.000**	**32.91**	**0.000**
**BMRQ**																				
Musical seeking	**15.26**	**0.000**	4.66	0.031	1.08	0.298	**24.59**	**0.000**	0.01	0.943	4.98	0.026	0.02	0.883	0.03	0.860	0.45	0.503	0.10	0.749
Emotion evocation	0.07	0.793	5.13	0.024	0.00	0.979	0.38	0.537	2.28	0.131	1.34	0.248	1.44	0.230	0.39	0.535	0.16	0.693	0.00	0.992
Emotion regulation	**8.25**	**0.004**	0.35	0.554	**16.57**	**0.000**	**11.97**	**0.001**	1.26	0.262	0.47	0.493	0.45	0.501	0.43	0.514	1.66	0.197	0.36	0.549
Sensory-motor	0.03	0.868	0.94	0.333	0.31	0.579	1.09	0.298	1.69	0.193	4.42	0.035	6.65	0.010	**11.49**	**0.001**	0.57	0.451	2.83	0.093
Social reward	6.92	0.009	**13.56**	**0.000**	4.37	0.036	4.91	0.027	0.76	0.382	**16.98**	**0.000**	**22.67**	**0.000**	**25.11**	**0.000**	0.32	0.569	5.75	0.017
**GSI**																				
Training	5.27	0.022	0.77	0.379	1.13	0.288	0.31	0.576	0.48	0.486	0.01	0.934	**25.93**	**0.000**	6.28	0.012	0.89	0.346	0.12	0.731
**ERQ**																				
Cognitive reappraisal	**12.35**	**0.000**	4.64	0.031	**10.43**	**0.001**	4.10	0.043	4.38	0.036	1.47	0.225	0.01	0.912	2.08	0.150	1.28	0.258	2.09	0.148
Emotional suppression	1.01	0.316	5.38	0.020	0.55	0.457	0.13	0.724	2.53	0.111	0.31	0.579	6.72	0.010	0.03	0.861	0.10	0.749	0.17	0.678
**SOCIODEMOGRAPHIC**																				
Age	0.96	0.326	**8.84**	**0.003**	0.25	0.619	0.53	0.467	0.09	0.761	0.02	0.903	0.39	0.534	0.08	0.775	0.09	0.766	5.57	0.018
Gender	**9.28**	0.010	1.76	0.416	0.87	0.649	0.26	0.877	0.49	0.785	0.38	0.826	3.08	0.214	7.21	0.027	4.54	0.103	3.46	0.178
Education	1.81	0.179	0.21	0.645	1.15	0.284	0.06	0.806	1.98	0.159	1.07	0.302	**10.99**	**0.001**	**11.64**	**0.001**	0.07	0.788	0.26	0.612

*Predictors with values found significant after applying Bonferroni correction are indicated in bold*.

Subjective reported changes in music listening in general during the crisis were positively associated with BMRQ facets of Musical Seeking [β = 0.07, SE = 0.019, $\chi_{\left(1\right)}^{2}$ = 15.26, *p*-corrected < 0.001], Emotion Regulation [β = 0.065, SE = 0.022, $\chi_{\left(1\right)}^{2}$ = 8.25, *p*-corrected = 0.04], and the ERQ Cognitive Reappraisal facet [β = 0.029, SE = 0.008, $\chi_{\left(1\right)}^{2}$ = 12.35, *p*-corrected = 0.004].

Regarding the distinction between happy and sad music, listening to happy music was specifically associated with individual differences in the BMRQ's Emotion Regulation facet [β = 0.082, SE = 0.020, $\chi_{\left(1\right)}^{2}$ = 16.57, *p*-corrected < 0.001], and with the Cognitive Reappraisal facet of the ERQ [β = 0.024, SE = 0.007, $\chi_{\left(1\right)}^{2}$ = 10.43, *p*-corrected = 0.012]. Those individuals that scored higher in these two facets reported seeking happy music much more during the pandemic than before. On the contrary, sad music was sought by individuals who scored high in Social Reward [β = 0.080, SE = 0.022, $\chi_{\left(1\right)}^{2}$ = 13.56, *p*-corrected = 0.002] and younger participants, as reflected by a significant negative relationship with age [β = −0.012, SE = 0.004, $\chi_{\left(1\right)}^{2}$ = 8.84, *p*-corrected = 0.030].

On the other hand, looking for novel music during the pandemic was positively associated with BMRQ facets of Musical Seeking [β = 0.092, SE = 0.019, $\chi_{\left(1\right)}^{2}$ = 8.84, *p*-corrected < 0.001] and Emotion Regulation [β = 0.078, SE = 0.023, $\chi_{\left(1\right)}^{2}$ = 8.84, *p*-corrected = 0.006]. Listening to music from childhood was not associated with any music reward facet or emotion regulation strategy. Finally, attendance to virtual concerts was positively associated with individual differences in BMRQ facet of Social Reward [β = 0.092, SE = 0.022, $\chi_{\left(1\right)}^{2}$ = 16.98, *p*-corrected < 0.001]. No significant relationship was found between musical training and engagement in musical listening activities (all *p*s > 0.05).

Regarding music production, engagement in singing and dancing activities during the lockdown were not associated with any music reward facet or emotion regulation strategy. However, engagement in playing and composing music was positively associated with the BMRQ facet of Social Reward [playing: β = 0.101, SE = 0.021, $\chi_{\left(1\right)}^{2}$ = 22.17, *p*-corrected < 0.001; composing: β = 0.108, SE = 0.021, $\chi_{\left(1\right)}^{2}$ = 25.11, *p*-corrected < 0.001]. In addition, musical training, as expected, was positively correlated with playing an instrument [β = 0.022, SE = 0.005, $\chi_{\left(1\right)}^{2}$ = 16.61, *p*-corrected < 0.001]. Finally, educational level was positively associated with engagement in playing [β = 0.213, SE = 0.064, $\chi_{\left(1\right)}^{2}$ = 11.17, *p*-corrected = 0.008] and composing music during the lockdown [β = 0.216, SE = 0.063, $\chi_{\left(1\right)}^{2}$ = 11.64, *p*-corrected = 0.006].

**Figure 2:**
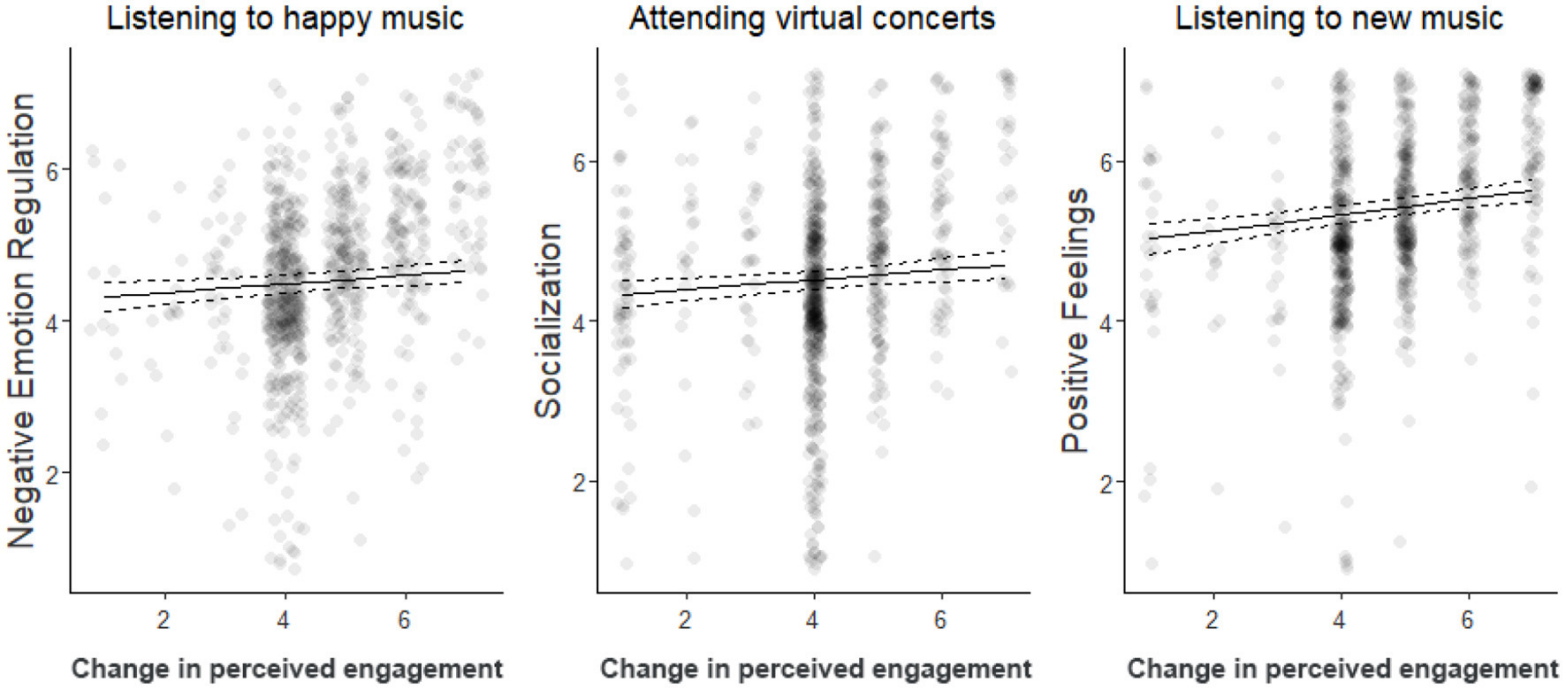
Main results (with 95% confidence interval) of the linear mixed-effects models estimating whether individuals used music as a way to regulate negative emotions (Negative Emotion Regulation scale), compensate the lack of social interaction (Socialization scale), or experience positive feelings (Positive Feelings scale) as function of the their engagement in listening to happy music, attending virtual concerts, and listening to new music, respectively. Each dot represents the response of one participant (a small jitter was applied for visualization purposes).

Another question is what people sought in these different music-related activities: to replace the lack of social interaction, regulate stress, or cheer them up? For that reason, we asked participants to indicate how engaging in music-related activities helped them cope with the psychological distress induced by the COVID-19 pandemic. Particularly, we assessed whether music-related activities helped them to (1) handle the current situation (“negative emotion regulation” scale), (2) reduce the feeling of social isolation (“socialization” scale), or (3) to make them feel good, relaxed, etc. (“positive feelings” scale). We then looked at the relationship between these three regulatory strategies and individuals' engagement in different music-related activities (Figure 2). Those who sought music-related activities to regulate and mitigate negative emotions listened to happy music much more than before, as reflected by a significant main effect of happy music on the “negative emotion regulation” scale [β = 0.057, SE = 0.021, $\chi_{\left(1\right)}^{2}$ = 6.98, *p*-corrected = 0.024]. Individuals who used music to overcome the lack of social interaction (“socialization” scale) reported greater changes in attending virtual concerts [β = 0.060, SE = 0.023, $\chi_{\left(1\right)}^{2}$ = 6.86, *p*-corrected = 0.027]. In addition, individuals with a higher education level tended to score higher in this scale too [β = 0.089, SE = 0.036, $\chi_{\left(1\right)}^{2}$ = 6.13, *p*-corrected = 0.040]. Finally, individuals who used music to amuse themselves (“positive feelings” scale) reported listening to music more in general [β = 0.053, SE = 0.020, $\chi_{\left(1\right)}^{2}$ = 7.26, *p*-corrected = 0.011], particularly seeking new music [β = 0.10, SE = 0.021, $\chi_{\left(1\right)}^{2}$ = 22.36, *p*-corrected < 0.001], but composed less [β = −0.073, SE = 0.024, $\chi_{\left(1\right)}^{2}$ = 9.13, *p*-corrected = 0.007] during the pandemic than before.

**Figure 3:**
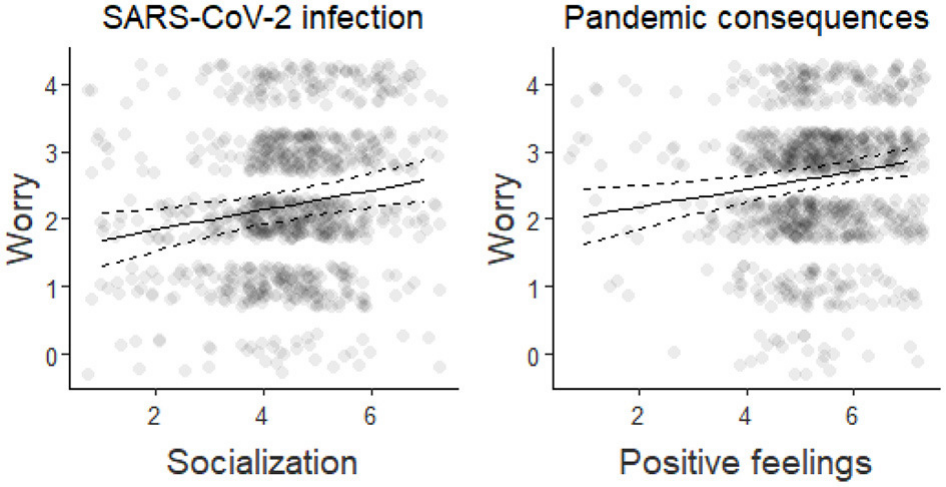
Main results (with 95% confidence interval) of the linear mixed-effects models estimating individuals' worries about the possibility of getting infected with the COVID-19, themself or their relatives/friends, or the consequence of the pandemic as function of their use of music to reduce social isolation or experience positive feelings, respectively. Each dot represents the response of one participant (a small jitter was applied for visualization purposes).

Next, we examined whether differences in these three regulatory strategies (negative emotion regulation, socialization, positive feelings) were reflecting individuals' worries about (i) the potential implications of the COVID-19 emergency in the future and (ii) the possibility of contracting the new coronavirus themselves or their loved ones. Those individuals who were more worried about the potential consequences of the COVID-19 crisis engaged in music-related activities to experience positive feelings [“positive feelings”: β = 0.134, SE = 0.044, $\chi_{\left(1\right)}^{2}$ = 9.28, *p*-corrected = 0.005]. On the contrary, those who were more concerned about the risk of contracting the COVID-19 virus (themselves or their relatives/friends) were more likely to engage in music-related activities as means of coping with the lack of social interactions [“socialization”: β = 0.145, SE = 0.047, $\chi_{\left(1\right)}^{2}$ = 9.69, *p*-corrected = 0.004; Figure 3].

## Discussion

Music is commonly used across cultures to achieve and maintain a positive affective state (Juslin and Vastfjall, 2008; Rentfrow, 2012; Zatorre and Salimpoor, 2013). People singing and playing from their balconies during the COVID-19 lockdown showed to the entire world how music could be used as strategy for facing a crisis (Mas-Herrero et al., 2020; Fink et al., 2021). But did our relationship with music change during the pandemic, and how? In this study, we aimed at investigating whether Covid-19 confinement modulated people's musical habits and whether this change was associated with individuals' musical and psychological traits and attempts to cope with specific worries and challenges related to the pandemic. The appropriate selection of musical materials, along with an appropriate strategy for affect regulation, has been shown to be important in the efficiency of the regulation of psychological distress (e.g., stress; Baltazar et al., 2019). Overall, our results showed (i) that during the confinement (May–June 2020), people were more engaged in musical activities involving music listening than before, regardless of individual differences (except for attending virtual concerts); (ii) that this change in behavior depended on individual differences in participants' sensitivity to musical reward, musical training, and emotional regulation strategies; and (iii) that individuals' engagement in different music-related activities was also associated with the purpose for which they engaged in music as a means for coping with the psychological distress induced by the pandemic: as a way to feel good, regulate their negative feelings, or feel connected to others. Our findings arise from participants' self-reports and memories of pre-pandemic times. The interpretation of these results should therefore bear in mind the lack of objective measures and retrospective follow-up.

A first important finding is that people were more engaged in certain musical activities during the confinement than before (Mas-Herrero et al., 2020; Cabedo-Mas et al., 2021; Fink et al., 2021). Overall, participants listened to music more, specifically happy music (but not sad), and regardless of its familiarity (i.e., they listened more to happy music, whether it was novel to them or from their childhood). We recently reported (Mas-Herrero et al., 2020) that, among many daily rewarding activities, people picked music most often as the best activity to cope with psychological distress related to the Covid-19 pandemic. Here, we extend this finding by showing that the perceived change in music listening specifically concerned positively valenced music. Happy music is an efficient way to induce happiness while reducing listeners' sadness, potentially through emotional contagion mechanisms (Lundqvist et al., 2009). Furthermore, happy music, as compared to sad music, has been shown to promote less self-reflection and self-related thoughts, as elicited while mind wandering (Taruffi et al., 2017). Therefore, an increase in positively valenced music listening might have helped participants to cope with the psychological distress induced by the pandemic, by evoking or maintaining positive affect while at the same time keeping individuals away from internal negative feelings (Taruffi and Koelsch, 2014). Indeed, individuals who sought happy music more during the pandemic than before also reported relying on music to regulate and mitigate their worries about the pandemic's negative emotional impact. They were also more prone to use music as a tool to regulate their mood and tended to use cognitive reappraisal strategies to reduce negative affect.

In this regard, previous studies have shown that engaging with music for the purpose of emotion regulation is associated with increased psychological well-being and reduced stress levels (Västfjäll et al., 2012; Helsing et al., 2016). Such an effect is possibly mediated by the habitual use of cognitive reappraisal (Chin and Rickard, 2014). Indeed, a series of studies focusing on the use of music for mood regulation showed that the majority of strategies aimed toward reducing- or diverting away from- negative feelings, as well as those aiming at the enhancement of positive state, were positively correlated with habitual use of cognitive reappraisal (Saarikallio, 2008, 2012). Here, we show that listening to happy music, but also the seeking of novel music, are associated with cognitive reappraisal emotional regulation strategies. That is, the ability to reinterpret an emotion-eliciting situation to change its emotional impact (Gross and John, 2003)—considered an efficient and healthy way to regulate emotions (Cutuli, 2014)—leads individuals to choose novel and positively valenced music. In addition, those individuals who sought new music more during the pandemic used music to elicit a positive mood, which may have helped them cope with their concerns about the potential implications of this crisis. Taken together, our findings point to a special role of seeking new music and engaging with-happy music as effective strategies to cope with the psychological distress induced by the pandemic (Saarikallio and Erkkilä, 2007).

As social isolation related to quarantine periods or confinement constitutes one of the pandemic's major issues, severely affecting people's mental health (Usher et al., 2020), it is particularly relevant to discuss the role played by the social aspects of music. We found the BMRQ *social reward* facet was positively associated with perceived change in engaging in different types of musical activities, particularly those involving music production (playing and composing) and attending virtual concerts. While the creative process might be seen as intimate, music composition could still be related to social (e.g., collective or audience-generated) aspects connecting the composer with others (see e.g., Collins, 2016). Furthermore, playing music and participating in concerts have been shown to strongly promote social connection (see e.g., Savage et al., 2020). Therefore, and in line with our results, these activities might been have used as a means to cope with the social isolation (see also Granot et al., 2021; MacDonald et al., 2021). Interestingly, we also found that individuals who scored higher in the “socialization” coping scale were those who reported greater changes in the frequency with which they attended virtual concerts, indicating that participating in collective virtual musical events helped people feel less lonely. These findings support the theory that music adaptively promotes social bonding (Cross, 2001; Savage et al., 2020) and suggest that it can be an effective social surrogate able to reduce loneliness (Schäfer and Eerola, 2020). Intriguingly, the BMRQ's Social Reward facet was also associated with listening to sad music. Previous findings indicate that sadness evoked by music may be particularly rewarding when it produces empathic feelings, caused, for example, by reflection on past or current events (Sachs et al., 2015). Notably, trait empathy has been associated with a preference for sad music, further confirming that empathetic engagement may play a relevant role in sadness-induced pleasure (Vuoskoski et al., 2012; Taruffi et al., 2021). Thus, individuals more prone to connect with others through music may have a particular predisposition to empathize and, therefore, to derive reward from sad music. In these individuals, sad music may be particularly helpful to cope with social isolation and feelings of sorrow.

While in general (i.e., regardless of individual differences) some music production activities (such as dancing or composing) decreased during the pandemic, probably due to the social restrictions, participants reported a significant increase in singing. Although our survey did not specifically investigate the type of singing activities (e.g., alone, with other members of the family, online; see also Theorell et al., 2020), these findings are in line with several studies highlighting that engaging in singing activities can be an excellent tool for coping, effectively increasing positive feelings, reducing stress, and promoting social connection in normal and clinical populations (Gick, 2011; Osman et al., 2016; Williams et al., 2018). Furthermore, despite the general trend, individuals who were particularly sensitive to the social aspects of music reward were more prone to engage in music production activities. Indeed, playing an instrument is generally reported as a way to reduce loneliness (North et al., 2000) and, therefore, may be an efficient strategy to overcome the lack of social interactions (see also Granot et al., 2021). It is also noteworthy that the effects discussed above were not specifically driven by people's musical expertise, although musical training was associated with an increase in playing an instrument. This would indicate that, while skilled musical behavior positively influences the engagement in musical production activities (Müllensiefen et al., 2014), music listening or singing could act as an effective emotional regulation strategy also in musically untrained individuals (Mas-Herrero et al., 2020).

In sum, our results revealed that, during the pandemic, individuals reported an overall increase in the frequency in which they engaged in certain music-related activities. Among all activities, listening to new and happy music were particularly associated with healthier emotion regulation strategies. Interestingly, while musical training only partially influenced individuals' changes in musical production activities, we found that different facets of musical reward and general emotional regulation strategies broadly modulated engagement in music-related activities during the pandemic in different directions. We thus provide converging evidence that music can be employed as an accessible effective, non-invasive, inter-cultural tool for helping people in dealing with their worries, feeling better, and reducing social isolation (Mas-Herrero et al., 2020; Cabedo-Mas et al., 2021; Fink et al., 2021; Granot et al., 2021). Furthermore, our data suggest that by employing psychometric measures such as musical hedonia scores (Mas-Herrero et al., 2013), we can highlight whether, how, and with whom music could be particularly powerful tool. Our results, thus, have broad implications for educational and clinical endeavors in which specific musical activities are tailored to each individual psychological profile, potentially leading to more fine-grained, personalized, and suitable interventions.

## Data Availability Statement

The raw data supporting the conclusions of this article will be made available by the authors, without undue reservation.

## Ethics Statement

The studies involving human participants were reviewed and approved by New York University's Committee on Activities Involving Human Subjects. The patients/participants provided their written informed consent to participate in this study.

## Author Contributions

LF, NS, PR, RZ, and EM-H designed the research. PR acquired the data. EM-H analyzed the data. LF, NS, and EM-H drafted the manuscript. LF, NS, MM, PR, RZ, and EM-H wrote the manuscript. All authors contributed to the article and approved the submitted version.

## Conflict of Interest

The authors declare that the research was conducted in the absence of any commercial or financial relationships that could be construed as a potential conflict of interest.

## Footnotes

^1^https://www.nytimes.com/interactive/2020/11/23/magazine/yo-yo-ma-interview.html

## References

[B1] Baltazar M. Västfjäll D. Asutay E. Koppel L. Saarikallio S. (2019). Is it me or the music? Stress reduction and the role of regulation strategies and music. Music Sci. 2:2059204319844161. 10.1177/2059204319844161

[B2] Balzarotti S. (2019). The emotion regulation questionnaire: factor structure and measurement invariance in an Italian sample of community dwelling adults. Curr. Psychol. 1–12. Available online at: https://link.springer.com/article/10.1007%2Fs12144-019-00426-3

[B3] Cabedo-Mas A. Arriaga-Sanz C. Moliner-Miravet L. (2021). Uses and perceptions of music in times of COVID-19: a Spanish population survey. Front. Psychol. 11:606180. 10.3389/fpsyg.2020.606180 33510681 PMC7835488

[B4] Cabello R. Salguero J. M. Fernández-Berrocal P. Gross J. J. (2013). A Spanish adaptation of the Emotion Regulation Questionnaire. Eur. J. Psychol. Assessment 29:234. Available online at: https://psycnet.apa.org/record/2013-38005-00230071082

[B5] Carlson E. Wilson J. Baltazar M. Duman D. Peltola H.-R. Toiviainen P.. (2021). The role of music in everyday life during the first wave of the coronavirus pandemic: a mixed-methods exploratory study. Front. Psychol. 12:647756. 10.3389/fpsyg.2021.647756 34017286 PMC8129180

[B6] Chapman L. J. Chapman J. P. Raulin M. L. (1976). Scales for physical and social anhedonia. J. Abnor. Psychol. 85, 374–382. 10.1037/0021-843X.85.4.374 956504

[B7] Chin T. Rickard N. S. (2014). Emotion regulation strategy mediates both positive and negative relationships between music uses and well-being. Psychol. Music 42, 692–713. 10.1177/0305735613489916

[B8] Clift S. Hancox G. Morrison I. Hess B. Kreutz G. Stewart D. (2010). Choral singing and psychological wellbeing: quantitative and qualitative findings from English choirs in a cross-national survey. J. Appl. Arts Health 1, 19–34. 10.1386/jaah.1.1.19/1

[B9] Collins D. (editor). (2016). The Act of Musical Composition: Studies in the Creative Process. Routledge. Available online at: https://books.google.es/books?hl=it&lr=&id=KXzeCwAAQBAJ&oi=fnd&pg=PP1&dq=collins+2016+music&ots=aeSIpOqitM&sig=gTpfdL3lfaNMsoOvs8Z2OMV0aYg&redir_esc=y#v=onepage&q=collins%202016%20music&f=false

[B10] Cross I. (2001). Music, cognition, culture, and evolution. Ann. N. Y. Acad. Sci. 930, 28–42. 10.1111/j.1749-6632.2001.tb05723.x 11458835

[B11] Cutuli D. (2014). Cognitive reappraisal and expressive suppression strategies role in the emotion regulation: an overview on their modulatory effects and neural correlates. Front. Syst. Neurosci. 8:175. 10.3389/fnsys.2014.00175 25285072 PMC4168764

[B12] D'Ausilio A. Novembre G. Fadiga L. Keller P. E. (2015). What can music tell us about social interaction? Trends Cogn. Sci. 19, 111–114. 10.1016/j.tics.2015.01.005 25641075

[B13] Fink L. Warrenburg L. A. Howlin C. Randall W. M. Hansen N. C. Wald-Fuhrmann M. (2021). Viral tunes: changes in musical behaviours and interest in coronamusic predict socio-emotional coping during COVID-19 lockdown. PsyArXiv [Preprint]. 10.31234/osf.io/7mg2v

[B14] Gibbs H. Egermann H. (2021). Music-evoked nostalgia and wellbeing during the United Kingdom COVID-19 pandemic: content, subjective effects, and function. Front. Psychol. 12:647891. 10.3389/fpsyg.2021.647891 33828512 PMC8019926

[B15] Gick M. L. (2011). Singing, health and well-being: a health psychologist's review. Psychomusicology 21, 175–207. 10.1037/h0094011

[B16] Granot R. Spitz D. H. Cherki B. R. Loui P. Timmers R. Schaefer R. S.. (2021). “Help! I need somebody”: music as a global resource for obtaining wellbeing goals in times of crisis. Front. Psychol. 12:648013. 10.3389/fpsyg.2021.648013 33935907 PMC8079817

[B17] Gross J. J. John O. P. (2003). Individual differences in two emotion regulation processes: implications for affect, relationships, and well-being. J. Pers. Soc. Psychol. 85, 348–362. 10.1037/0022-3514.85.2.348 12916575

[B18] Helsing M. Västfjäll D. Bjälkebring P. Juslin P. Hartig T. (2016). An experimental field study of the effects of listening to self-selected music on emotions, stress, and cortisol levels. Music Med. 8, 187–198. 10.47513/mmd.v8i4.442

[B19] Janata P. (2009). The neural architecture of music-evoked autobiographical memories. Cereb. Cortex 19, 2579–2594. 10.1093/cercor/bhp008 19240137 PMC2758676

[B20] Janata P. Tomic S. T. Rakowski S. K. (2007). Characterisation of music-evoked autobiographical memories. Memory 15, 845–860. 10.1080/09658210701734593 17965981

[B21] John O. P. Gross J. J. (2004). Healthy and unhealthy emotion regulation: personality processes, individual differences, and life span development. J. Pers. 72, 1301–1334. 10.1111/j.1467-6494.2004.00298.x 15509284

[B22] Juslin P. N. Vastfjall D. (2008). Emotional responses to music: the need to consider underlying mechanisms. Behav. Brain Sci. 31, 559–575; discussion: 575–621. 10.1017/S0140525X08005293 18826699

[B23] Koelsch S. (2013). From social contact to social cohesion–the 7 Cs. Music Med. 5, 204–209. 10.1177/1943862113508588

[B24] Larsen R. J. (2000). Toward a science of mood regulation. Psychol. Inquiry 11, 129–141. 10.1207/S15327965PLI1103_01

[B25] Laukka P. (2007). Uses of music and psychological well-being among the elderly. J. Happiness Stud. 8, 215–241. 10.1007/s10902-006-9024-319445633

[B26] Livingstone K. M. Srivastava S. (2012). Up-regulating positive emotions in everyday life: strategies, individual differences, and associations with positive emotion and well-being. J. Res. Pers. 46, 504–516. 10.1016/j.jrp.2012.05.009

[B27] Lonsdale A. J. North A. C. (2009). Musical taste and ingroup favouritism. Group Proc. Intergroup Relat. 12, 319–327. 10.1177/1368430209102842

[B28] Lonsdale A. J. North A. C. (2011). Why do we listen to music? A uses and gratifications analysis. Br. J. Psychol. 102, 108–134. 10.1348/000712610X506831 21241288

[B29] Lundqvist L.-O. Carlsson F. Hilmersson P. Juslin P. N. (2009). Emotional responses to music: experience, expression, and physiology. Psychol. Music 37, 61–90. 10.1177/0305735607086048

[B30] MacDonald R. Burke R. De Nora T. Sappho Donohue M. Birrell R. (2021). Our virtual tribe: sustaining and enhancing community via online music improvisation. Front. Psychol. 11:623640. 10.3389/fpsyg.2020.623640 33708151 PMC7940664

[B31] MacDonald R. Kreutz G. Mitchell L. (2013). Music, Health, and Wellbeing. Oxford: Oxford University Press.

[B32] Martín J. C. Ortega-Sánchez D. Miguel I. N. Gil Martín G. M. (2021). Music as a factor associated with emotional self-regulation: a study on its relationship to age during COVID-19 lockdown in Spain. Heliyon 7:e06274. 10.1016/j.heliyon.2021.e06274 33665439 PMC7907216

[B33] Martínez-Castilla P. Gutiérrez-Blasco I. M. Spitz D. H. Granot R. (2021). The efficacy of music for emotional wellbeing during the COVID-19 lockdown in Spain: an analysis of personal and context-related variables. Front. Psychol. 12:647837. 10.3389/fpsyg.2021.647837 33897554 PMC8062927

[B34] Martínez-Molina N. Mas-Herrero E. Rodríguez-Fornells A. Zatorre R. J. Marco-Pallarés J. (2016). Neural correlates of specific musical anhedonia. Proc. Natl. Acad. Sci. U.S.A. 113, E7337–E7345. 10.1073/pnas.1611211113 27799544 PMC5135354

[B35] Mas-Herrero E. Marco-Pallares J. Lorenzo-Seva U. Zatorre R. J. Rodriguez-Fornells A. (2013). Individual differences in music reward experiences. Music Percept. 31, 118–138. 10.1525/mp.2013.31.2.118

[B36] Mas-Herrero E. Singer N. Ferreri L. McPhee M. Zatorre R. Ripolles P. (2020). Rock'n' Roll but not sex or drugs: music is negatively correlated to depressive symptoms during the COVID-19 pandemic via reward-related mechanisms. PsyArXiv [Preprint]. 10.31234/osf.io/x5upn 36401802

[B37] Mazza C. Ricci E. Biondi S. Colasanti M. Ferracuti S. Napoli C.. (2020). A nationwide survey of psychological distress among Italian people during the COVID-19 pandemic: immediate psychological responses and associated factors. Int. J. Environ. Res. Public Health 17:3165. 10.3390/ijerph17093165 32370116 PMC7246819

[B38] Moore S. A. Zoellner L. A. Mollenholt N. (2008). Are expressive suppression and cognitive reappraisal associated with stress-related symptoms? Behav. Res. Ther. 46, 993–1000. 10.1016/j.brat.2008.05.001 18687419 PMC2629793

[B39] Müllensiefen D. Gingras B. Musil J. Stewart L. (2014). The musicality of non-musicians: an index for assessing musical sophistication in the general population. PLoS ONE 9:e89642. 10.1371/journal.pone.0089642 24586929 PMC3935919

[B40] North A. C. Hargreaves D. J. O'Neill S. A. (2000). The importance of music to adolescents. Br. J. Educ. Psychol. 70, 255–272. 10.1348/000709900158083 10900782

[B41] Osman S. E. Tischler V. Schneider J. (2016). ‘Singing for the brain': a qualitative study exploring the health and well-being benefits of singing for people with dementia and their carers. Dementia 15, 1326–1339. 10.1177/1471301214556291 25425445 PMC5089222

[B42] Páez D. Martínez-Sánchez F. Mendiburo A. Bobowik M. Sevillano V. (2013). Affect regulation strategies and perceived emotional adjustment for negative and positive affect: a study on anger, sadness and joy. J. Positive Psychol. 8, 249–262. 10.1080/17439760.2013.786751

[B43] Rentfrow P. J. (2012). The role of music in everyday life: current directions in the social psychology of music. Soc. Pers. Psychol. Compass 6, 402–416. 10.1111/j.1751-9004.2012.00434.x

[B44] Saarikallio S. (2008). Music in mood regulation: initial scale development. Musicae Sci. 12, 291–309. 10.1177/102986490801200206

[B45] Saarikallio S. (2012). Development and validation of the brief music in mood regulation scale (B-MMR). Music Percept. Interdisc. J. 30, 97–105. 10.1525/mp.2012.30.1.97

[B46] Saarikallio S. Erkkilä J. (2007). The role of music in adolescents' mood regulation. Psychol. Music 35, 88–109. 10.1177/0305735607068889

[B47] Sachs M. E. Damasio A. Habibi A. (2015). The pleasures of sad music: a systematic review. Front. Hum. Neurosci. 9:404. 10.3389/fnhum.2015.00404 26257625 PMC4513245

[B48] Savage P. E. Loui P. Tarr B. Schachner A. Glowacki L. Mithen S.. (2020). Music as a coevolved system for social bonding. Behav. Brain Sci. 1–36. 10.1017/S0140525X20000333 32814608

[B49] Schäfer K. Eerola T. (2020). How listening to music and engagement with other media provide a sense of belonging: an exploratory study of social surrogacy. Psychol. Music 48, 232–251. 10.1177/0305735618795036

[B50] Schäfer K. Saarikallio S. Eerola T. (2020). Music may reduce loneliness and act as social surrogate for a friend: evidence from an experimental listening study. Music Sci. 3:205920432093570. 10.1177/2059204320935709

[B51] Schulkind M. D. Hennis L. K. Rubin D. C. (1999). Music, emotion, and autobiographical memory: they're playing your song. Mem. Cogn. 27, 948–955. 10.3758/BF03201225 10586571

[B52] Sloboda J. A. O'Neill S. A. Ivaldi A. (2001). Functions of music in everyday life: an exploratory study using the experience sampling method: Musicae Sci. 5, 9–32. 10.1177/102986490100500102

[B53] Taruffi L. Koelsch S. (2014). The paradox of music-evoked sadness: an online survey. PLoS ONE 9:e110490. 10.1371/journal.pone.0110490 25330315 PMC4203803

[B54] Taruffi L. Pehrs C. Skouras S. Koelsch S. (2017). Effects of sad and happy music on mind-wandering and the default mode network. Sci. Rep. 7:14396. 10.1038/s41598-017-14849-0 29089542 PMC5663956

[B55] Taruffi L. Skouras S. Pehrs C. Koelsch S. (2021). Trait empathy shapes neural responses toward sad music. Cogn. Affect. Behav. Neurosci. 21, 231–241. 10.3758/s13415-020-00861-x 33474716 PMC7994216

[B56] Theorell T. Kowalski J. Theorell A. M. L. Horwitz E. B. (2020). Choir singers without rehearsals and concerts? A questionnaire study on perceived losses from restricting choral singing during the covid-19 pandemic. J. Voice. 10.1016/j.jvoice.2020.11.006 33288380

[B57] Usher K. Durkin J. Bhullar N. (2020). The COVID-19 pandemic and mental health impacts. Int. J. Ment. Health Nurs. 29, 315–318. 10.1111/inm.12726 32277578 PMC7262128

[B58] Västfjäll D. Juslin P. N. Hartig T. others (2012). Music, subjective wellbeing, and health: the role of everyday emotions, in Music, Health and Wellbeing, eds MacDonald R. A. R. Kreutz G. Mitchell L. (Oxford University Press), 405–423. 10.1093/acprof:oso/9780199586974.003.0027

[B59] Vuoskoski J. K. Thompson W. F. McIlwain D. Eerola T. (2012). Who enjoys listening to sad music and why? Music Percept. 29, 311–317. 10.1525/mp.2012.29.3.311

[B60] Williams E. Dingle G. A. Clift S. (2018). A systematic review of mental health and wellbeing outcomes of group singing for adults with a mental health condition. Eur. J. Public Health 28, 1035–1042. 10.1093/eurpub/cky115 29982515

[B61] Witek M. A. G. Clarke E. F. Wallentin M. Kringelbach M. L. Vuust P. (2014). Syncopation, body-movement and pleasure in groove music. PLoS ONE 9:e94446. 10.1371/journal.pone.0094446 24740381 PMC3989225

[B62] Xiong J. Lipsitz O. Nasri F. Lui L. M. W. Gill H. Phan L.. (2020). Impact of COVID-19 pandemic on mental health in the general population: a systematic review. J. Affect. Disord. 277, 55–64. 10.1016/j.jad.2020.08.001 32799105 PMC7413844

[B63] Zatorre R. J. Salimpoor V. N. (2013). From perception to pleasure: music and its neural substrates. Proc. Natl. Acad. Sci. U.S.A. 110, 10430–10437. 10.1073/pnas.1301228110 23754373 PMC3690607

