# Supplementary material for: Engagement in Music-Related Activities During the COVID-19 Pandemic as a Mirror of Individual Differences in Musical Reward and Coping Strategies
Source: Front Psychol. 2021 Jun 28;12:673772. doi: 10.3389/fpsyg.2021.673772 (PMC8273332; doi:10.3389/fpsyg.2021.673772)
Supplement: Supplementary file 1 [file Data_Sheet_1.PDF]

Supplementary Material

Ferreri, L., Singer, N., McPhee, M., Ripollés, P., Zatorre, R.J., Mas-Herrero, E. **"Engagement in music-related activities during the COVID-19 pandemic as a mirror of individual differences in musical reward and coping strategies"**

Survey

Note that questionnaires (such as Physical Anhedonia Scale and Depression Anxiety Stress Scale 21) not considered in the current manuscript are not reported in the following. For non-musical activities investigated by the survey, see Mas-Herrero et al. (2020).

Instructions

You will be asked to complete several questionnaires designed to assess your demographic, linguistic, economic, and educational background, your personality, your attitude toward typically rewarding stimuli (e.g., food, music), and your attitude toward and ways of coping with a moment of crisis such as the one provoked by the new coronavirus (COVID-19) emergency. There are no right or wrong responses. Please provide honest answers.

(VOLUNTEERS SEE THIS)

It takes a maximum of 30-45 minutes to complete the entire survey. If you do not have enough time right now, no worries - you will be able return to the last page you left unfilled as long as you are using the same device (computer, tablet, o telephone) and browser. Leave the page open and come back to it later or click again on the link that you followed originally to get to this page. Your input is highly valuable and we appreciate your time and cooperation in filling this out!

Please, input an ID code. Your ID code is:

The first 2 letters of the first name of the person who raised you.

The first 2 letters of the city in which you lived when you were 10 years old.

Your age .

For example, if my mother's name is Mary, I lived in New York city when I was 10 years old and I am 34 years old, my ID would be 'MANE34'.

(MTURK PAID WORKERS SEE THIS)

Please,for the ID field, use a your MTURK worker ID:

ID:_____

Demographics

1. Gender:__________

2. Age: _____

3. Are you of Hispanic, Latino, or of Spanish origin? Yes or No

4. How would you describe yourself?

    - American Indian or Alaska Native
    - Asian
    - Black or African American
    - Native Hawaiian or Other Pacific Islander
    - White

5. Location of residence during the new coronavirus (COVID-19) emergency; include country and region (for example: USA/San Diego, California or France/Marseille):

    ____

6. Highest level of education completed:
    - ❑ Did not complete elementary school
    - ❑ Elementary school
    - ❑ High School
    - ❑ Bachelor's degree (e.g. B.A., B.Sc.)
    - ❑ Graduate studies (e.g. M.A., M.Sc., Ph.D., M.D.)

7. Employment status **before** the new coronavirus (COVID-19) emergency started (select all that apply):
    - ❑ Unemployed
    - ❑ Student

- ❏ Employed, part time
- ❏ Employed, full time
- ❏ Employed, multiple jobs
- ❏ Underemployed (I was having trouble finding as much work as I would like)
- ❏ On an unpaid leave of absence
- ❏ On a paid leave of absence
- ❏ Working from home
- ❏ Working more than usual
- ❏ Working less than usual
- ❏ Working as a healthcare professional
- ❏ Retired
- ❏ Self-employed

8. Annual household income **before** the new coronavirus (COVID-19) emergency started in USD:

   - ❏ Less than $10,000
   - ❏ $10,001 - $20,000
   - ❏ $20,001 - $50,000
   - ❏ $50,001 - $75,000
   - ❏ $75,001 - $150,000
   - ❏ $150,001 - $200,000
   - ❏ More than $200,001

   (ONLY FOR VOLUNTEERS)
   If you do not know the equivalence of your annual household income in dollars or euros, please write here your approximate annual income in your currency. For example, 'Between 100,000 and 200,000 Argentine pesos":_____

9. Marital status:

   - ❏ Married
   - ❏ Engaged
   - ❏ In a domestic partnership, cohabitation, or de facto marriage
   - ❏ Separated
   - ❏ Widowed
   - ❏ Divorced
   - ❏ Never married

10. Parental status (select all that apply):

    - ❏ Parent to one or more children under 18 years old
    - ❏ Parent to one or more children 18 years or older
    - ❏ No children

11. Number of individuals currently living in your household including yourself: _____

12. Number of children under 18 years old currently living in your household: _____

13. Number of adults at least 65 years old currently living in your household: _____

BMRQ

The following survey aks questions regarding your attitude towards music.Each item of this questionnaire is a statement that a person may either agree with or disagree with. For each item, indicate how much you agree or disagree with what the item says. Please respond to all the items; do not leave any blank. Choose only one response to each statement. Please be as accurate and honest as you can be. Respond to each item as if it were the only item. That is, do not worry about being consistent in your responses. Choose from strongly disagree to strongly agree one of the five options.

[1] Completely disagree; [2] Disagree; [3] Neither agree nor disagree; [4] Agree; [5] Completely agree.

	(1)	(2)	(3)	(4)	(5)
1. When I share music with someone, I feel a special connection with that person.	○	○	○	○	○
2. In my free time I hardly listen to music.	○	○	○	○	○
3. I like listening to music that contains emotion.	○	○	○	○	○
4. Music keeps me company when I'm alone.	○	○	○	○	○
5. I don't like to dance, not even with music I like.	○	○	○	○	○
6. Music makes me bond with other people.	○	○	○	○	○
7. I inform myself about music I like.	○	○	○	○	○
8. I get emotional listening to certain pieces of music.	○	○	○	○	○
9. Music calms and relaxes me.	○	○	○	○	○
10. Music often makes me dance.	○	○	○	○	○
11. I'm always looking for new music.	○	○	○	○	○
12. I can become tearful or cry when I listen to a melody that I like very much.	○	○	○	○	○
13. I like to sing or play an instrument with other people.	○	○	○	○	○
14. Music helps me chill out.	○	○	○	○	○
15. I can't help humming or singing along to music that I like.	○	○	○	○	○
16. At a concert I feel connected to the performers and the audience.	○	○	○	○	○
17. I spend quite a bit of money on music and related items.	○	○	○	○	○
18. I sometimes feel chills when I hear a melody that I like.	○	○	○	○	○

19. Music comforts me.	○	○	○	○	○
20. When I hear a tune I like a lot, I can't help tapping or moving to its beat.	○	○	○	○	○

If you have doubts or are experiencing any technical difficulties, please contact us at **steinhardt-marl-ripolleslab@nyu.edu**

Here are some statements about attitudes and experiences. Please mark each statement as true or false to describe your own attitudes and experiences. We want you to describe yourself as you have been during most of your adult life. Please mark every statement, even if you are not quite sure about the answer:

The following survey asks questions regarding your experience with music. Some of the items of this questionnaire are statements that a person may either agree with or disagree with. For each item, indicate how much you agree or disagree with what the item says. Some of them ask about your specific experience with music (for example, years playing a musical instrument). Please respond to all the items; do not leave any blank. Provide only one response to each statement. Please be as accurate and honest as you can be. Respond to each item as if it were the only item. That is, don't worry about being consistent in your responses.

Please answer these questions with your attitudes and experiences BEFORE THE NEW CORONAVIRUS (COVID-19) EMERGENCY HAPPENED

1. Completely disagree
2. Strongly disagree
3. Disagree
4. Neither agree nor disagree
5. Agree
6. Strongly agree
7. Completely agree

	(1)	(2)	(3)	(4)	(5)	(6)	(7)
1. I spend a lot of my free time doing music-related activities.	○	○	○	○	○	○	○
2. I enjoy writing about music, for example on blogs and forums.	○	○	○	○	○	○	○
3. If somebody starts singing a song I don't know, I can usually join in.	○	○	○	○	○	○	○
4. I am able to judge whether someone is a good singer or not.	○	○	○	○	○	○	○
5. I usually know when I'm hearing a song for the first time.	○	○	○	○	○	○	○

6. I can sing or play music from memory.

7. I am able to hit the right notes when I sing along with a recording.

8. I find it difficult to spot mistakes in a performance of a song even if I know the tune.

9. I can compare and discuss differences between two performances or versions of the same piece of music.

10. I have trouble recognizing a familiar song when played in a different way or by a different performer.

11. I have never been complimented for my talents as a musical performer.

12. I often read or search the internet for things related to music.

13. I am not able to sing in harmony when somebody is singing a familiar tune.

14. I can tell when people sing or play out of time with the beat.

15. I am able to identify what is special about a given musical piece.

16. I can tell when people sing or play out of tune.

17. When I sing, I have no idea whether I'm in tune or not.

18. Music is kind of an addiction for me - I couldn't live without it.

19. I don't like singing in public because I'm afraid that I would sing wrong notes.

20. When I hear a piece of music I can usually identify its genre.

21. I would not consider myself a musician.

22. After hearing a new song two or three times, I can usually sing it by myself.

23. I engaged in regular, daily practice of a musical instrument (including voice) for :

- 0 years
- 1 years
- 2 years
- 3 years
- 4-5 years
- 6-9 years

- 10 or more years

24. At the peak of my interest, I practiced :
- 0 hours per day on my primary instrument
- 0.5 hours per day on my primary instrument (including voice)
- 1 hours per day on my primary instrument (including voice)
- 1.5 hours per day on my primary instrument (including voice)
- 2 hours per day on my primary instrument (including voice)
- 3-4 hours per day on my primary instrument (including voice)
- 5 hours per day on my primary instrument (including voice)

25. I have had formal training in music theory for :
- 0 years
- 0.5 years
- 1 years
- 2 years
- 3 years
- 4-6 years
- 7 or more years

26. I have had :
- 0 years of formal training on a musical instrument (including voice) during my lifetime
- 0.5 years of formal training on a musical instrument (including voice) during my lifetime
- 1 years of formal training on a musical instrument (including voice) during my lifetime
- 2 years of formal training on a musical instrument (including voice) during my lifetime
- 3-5 years of formal training on a musical instrument (including voice) during my lifetime
- 6-9 years of formal training on a musical instrument (including voice) during my lifetime
- 10 or more years of formal training on a musical instrument (including voice) during my lifetime

27. I can play :
- 0 musical instruments
- 1 musical instruments
- 2 musical instruments
- 3 musical instruments
- 4 musical instruments
- 5 musical instruments
- 6 or more musical instruments

28. If you had formal musical training, how old were you when it started? Input 0 if you did not receive formal training: _____

29. I listen attentively to music for :
- 0-15 min per day
- 15-30 min per day

- 30-60 min per day
- 60-90 min per day
- 2 hours per day
- 2-3 hours per day
- 4 hours or more per day

30. What kinds of music do you listen to (pop, rock, classic, jazz, rap, latin,etc)? If multiple kinds, list in order of preference :

____

ERQ

We would like to ask you some questions about your emotional life, in particular, how you control (that is, regulate and manage) your emotions. The questions below involve two distinct aspects of your emotional life. One is your emotional experience, or what you feel like inside. The other is your emotional expression, or how you show your emotions in the way you talk, gesture, or behave. Although some of the following questions may seem similar to one another, they differ in important ways.

Please answer these questions with your attitudes and experiences BEFORE THE NEW CORONAVIRUS (COVID-19) EMERGENCY HAPPENED

1----------------2-----------------3-----------------4-----------------5-----------------6-----------------7
strongly disagree ***neutral*** ***strongly agree***

	(1)	(2)	(3)	(4)	(5)	(6)	(7)
1. When I want to feel more *positive* emotion (such as joy or amusement), I *change what I'm thinking about*.	○	○	○	○	○	○	○
2. I keep my emotions to myself.	○	○	○	○	○	○	○
3. When I want to feel less *negative* emotion (such as sadness or anger), I *change what I'm thinking about*.	○	○	○	○	○	○	○
4. When I am feeling *positive* emotions, I am careful not to express them.	○	○	○	○	○	○	○
5. When I'm faced with a stressful situation, I make myself *think about it* in a way that helps me stay calm.	○	○	○	○	○	○	○
6. I control my emotions by *not expressing them*.	○	○	○	○	○	○	○
7. When I want to feel more *positive* emotion, I *change the way I'm thinking* about the situation.	○	○	○	○	○	○	○
8. I control my emotions by *changing the way I think* about the situation I'm in.	○	○	○	○	○	○	○
9. When I am feeling *negative* emotions, I make sure	○	○	○	○	○	○	○

not to express them.

10. When I want to feel less *negative* emotion, I *change the way I'm thinking* about the situation.	◌	◌	◌	◌	◌	◌	◌

COVID-19 Survey

Please answer the following questions regarding how the new coronavirus (COVID-19) emergency has affected your lifestyle.

1. Are you currently diagnosed, or have been diagnosed with the new coronavirus (COVID-19)?
   - ❑ No
   - ❑ Yes
2. Even if you were not diagnosed (with a test), do you think you had the new coronavirus (COVID-19)?
   - ❑ No
   - ❑ Yes

3. Is someone close to you diagnosed, or has been diagnosed with new coronavirus (COVID-19)?
   - ❑ No
   - ❑ Yes

4. Did someone close to you pass away from new coronavirus (COVID-19) complications?
   - ❑ No
   - ❑ Yes

5. Have you made any changes to your lifestyle or daily activities because of the new coronavirus (COVID-19) emergency?
   - ❑ No
   - ❑ Yes

5b. Which of the following are you doing? (Select all that apply)
   - ❑ Washing hands more
   - ❑ Avoiding social gatherings
   - ❑ Stocking up on food and supplies
   - ❑ Avoiding or canceling domestic travel
   - ❑ Working from home
   - ❑ Avoiding gym and exercise classes
   - ❑ Avoiding or canceling international travel
   - ❑ Avoiding routine health care appointments
   - ❑ Not attending classes
   - ❑ I am making no changes to my lifestyle

6. Has your employment status changed because of the new coronavirus (COVID-19) emergency? (Select all that apply)
   - ❏ No
   - ❏ Yes, I have a reduced income
   - ❏ Yes, I have lost my job
   - ❏ Yes, my workplace went out of business
   - ❏ Yes, my working hours have been reduced
   - ❏ Yes, my working hours have been increased
   - ❏ Yes, my working conditions have become stressful
   - ❏ Yes, my job responsibilities have changed
   - ❏ Yes, I work from home

7. Do you belong to a new coronavirus (COVID-19) risk group?
   - ❏ Yes
   - ❏ No
   - ❏ I don’t know

8. How would you define your current health status?
   - ❏ Excellent
   - ❏ Very good
   - ❏ Good
   - ❏ Fair
   - ❏ Poor
   - ❏ I am not sure

9. Are you a healthcare professional?
   - ❏ Yes
   - ❏ No

10. Do you live with a healthcare professional?
    - ❏ Yes
    - ❏ No

11. Do you currently hold any form of health insurance?
    - ❏ No
    - ❏ Yes, I have a private health insurance
    - ❏ Yes, my country has a public health coverage
    - ❏ I don't know

12. Was your area of residence under confinement orders during the new coronavirus (COVID-19) emergency?
    - ❏ No
    - ❏ Yes
    - ❏ I don’t know

12b. For how long have you been confined during the new coronavirus (COVID-19) emergency? Input the number of weeks.

12c. Have confinement orders in your area of residence been lifted or partially lifted at this point in time? If so indicate for how many days (input 0 if the confinement orders are still in place).

13. Did/Do you encounter any of the following difficulties during the new coronavirus (COVID-19) emergency? Please indicated all that apply):
    - ❏ Getting food
    - ❏ Getting medications
    - ❏ Getting sanitizing and/or cleaning supplies
    - ❏ Using public transportation
    - ❏ Accessing healthcare
    - ❏ Increased childcare duties
    - ❏ Increased household duties (i.e., cleaning, etc.)
    - ❏ Social isolation and loneliness
    - ❏ Moved out of my home
    - ❏ I did not encounter any difficulties at all
    - ❏ Other (Write 'No' if you did not encounter any additional difficulties listed above):

14. To what extent are you self-isolating?
    - ❏ All of the time - I am staying at home nearly all the time
    - ❏ Most of the time - I only leave my home to buy food and other essentials.
    - ❏ Some of the time. I have reduced the amount of times I am in public spaces, social gatherings, or at work.
    - ❏ None of the time. I am doing everything I normally do.

15. How worried do you feel that you or your loved ones will contract the new coronavirus (COVID-19)?
    - ❏ Not at all worried
    - ❏ Slightly worried
    - ❏ Moderately worried
    - ❏ Very worried
    - ❏ Extremely worried

16. How worried are you about the implications of the new coronavirus (COVID-19) emergency?
    - ❏ Not at all worried
    - ❏ Slightly worried
    - ❏ Moderately worried
    - ❏ Very worried
    - ❏ Extremely worried

17. Can you indicate any hopeful sides or positive aspects that you have discovered during the new coronavirus (COVID-19) emergency?
    - ❑ ______

If you have doubts or are experiencing any technical difficulties, please contact us at **steinhardt-marl-ripolleslab@nyu.edu**

How has your engagement in the following activities changed since the onset of the **new coronavirus (COVID-19) emergency*?* Please read the following statements and indicate if you are engaging more or less in these activities.**

	**I prefer not to answer**	Much Less (1)	(2)	(3)	About the same (4)	(5)	(6)	Much more (7)
1. Listening to music I used to listen to when I was younger (childhood and/or adolescence)	○	○	○	○	○	○	○	◉
2. Listening to music	○	○	○	○	○	○	○	○
3. Listening to happy music	○	○	○	○	○	○	○	○
4. Listening to sad music	○	○	○	○	○	○	○	○
5. Discovering/searching for new music	○	○	○	○	○	○	○	○
6. Watching live or recorded concerts	○	○	○	○	○	○	○	○
7. Playing music	○	○	○	○	○	○	○	○
8. Composing/creating new music	○	○	○	○	○	○	○	○
9. Dancing	○	○	○	○	○	○	○	○
10. Singing	○	○	○	○	○	○	○	○
11. Sexual activities (alone or with others)	○	○	○	○	○	○	○	○
12. Watching pornographic or erotic films/pictures	○	○	○	○	○	○	○	○
13. Cooking my favourite/old meals	○	○	○	○	○	○	○	○
14. Cooking	○	○	○	○	○	○	○	○
15. Baking (bread, cakes, cookies, etc)	○	○	○	○	○	○	○	○

16. Discovering new meals/tastes/recipes	○	○	○	○	○	○	○	○
17. Eating food with high sugar/caloric content	○	○	○	○	○	○	○	○
18. Exercising indoors	○	○	○	○	○	○	○	○
19. Exercising outdoors	○	○	○	○	○	○	○	○
20. Looking for new ways to exercise	○	○	○	○	○	○	○	○
21. Engaging in social media	○	○	○	○	○	○	○	○
22. Looking for new ways to interact through social media (for example, using a new app)	○	○	○	○	○	○	○	○
23. Talking with friends/family/colleagues (by phone or by video call)	○	○	○	○	○	○	○	○
24. Learning a new skill or hobby	○	○	○	○	○	○	○	○
25. Playing games (table/video games, in person/online)	○	○	○	○	○	○	○	○
26. Watching series, movies or documentaries	○	○	○	○	○	○	○	○
27. Photography/ Video recordings/ Media creation	○	○	○	○	○	○	○	○
28. Reading (books, magazines, articles, etc)	○	○	○	○	○	○	○	○
29. Meditating<br>30. Practicing yoga	○	○	○	○	○	○	○	○
31. Religious activities (for example, praying)	○	○	○	○	○	○	○	○
32. Writing / journaling	○	○	○	○	○	○	○	○
33. Attending virtual museums or art galleries	○	○	○	○	○	○	○	○
34. Listening to radio or podcasts	○	○	○	○	○	○	○	○
35. Working	○	○	○	○	○	○	○	○
36. Consuming alcohol	○	○	○	○	○	○	○	○
37. Consuming drugs	○	○	○	○	○	○	○	○
38. Consuming pills/sedatives	○	○	○	○	○	○	○	○
39. Going to a therapist (in-person or online)	○	○	○	○	○	○	○	○
40. Reading books, I already read	○	○	○	○	○	○	○	○

41. Looking for new books to read	○	○	○	○	○	○	○	○
42. Watching movies/series/documentaries I already watched	○	○	○	○	○	○	○	○
43. Looking for new movies/series/documentaries	○	○	○	○	○	○	○	○

Which of the previous activities have helped you the most to cope with the* new coronavirus (COVID-19) emergency*? Please, pick the top three items from the list above and give an example (a song or a band you have listen to a lot, a food you have cooked regularly, a book or an author you have read that has been helpful, a video game you have played a lot, people you have called more, etc)

1st. (item number)
Example:

2nd. (item number)
Example:

3rd. (item number)
Example:

Is there any other activity that is not listed here that has helped you to cope with the current situation? Please, indicate which one in your own words:___________

***In the following sections we will ask you specific questions about how different activities helped you cope with the* new coronavirus (COVID-19) emergency. Each item of this questionnaire is a statement that a person may either agree with or disagree with. For each item, indicate how much you agree or disagree with what the item says using the options given. Please respond to all the items; do not leave any blank. Choose only one response to each statement. Please be as accurate and honest as you can be. Respond to each item as if it were the only item. That is, do not worry about being consistent in your responses.**

After the* new coronavirus (COVID-19) emergency *started and measures to contain the virus were introduced in your area (for example, confinement, social distancing, etc):

LISTENING TO MUSIC…

	Not applicable/	Completely	Strongly	Disagree	Neither agree	Agree	Strongly	Completely Agree

	**I prefer not to answer**	disagree		Disagre e		nor disagree	Agre e	
…helped me to deal with the current situation	○	○	○	○	○	○	○	○
…helped me to overcome the lack of social interaction	○	○	○	○	○	○	○	○
...gave me solace	○	○	○	○	○	○	○	○
…helped me to get rid of my worries	○	○	○	○	○	○	○	○
…kept me company	○	○	○	○	○	○	○	○
…comforted me	○	○	○	○	○	○	○	○
…made me feel I have certain control over the situation	○	○	○	○	○	○	○	○
…made me feel less lonely or isolated	○	○	○	○	○	○	○	○
…relaxed me	○	○	○	○	○	○	○	○
…made me forget about the current situation	○	○	○	○	○	○	○	○
...made me feel like I'm part of a group	○	○	○	○	○	○	○	○
...helped me to perk up	○	○	○	○	○	○	○	○
…made me feel like time is flying	○	○	○	○	○	○	○	○
…made me feel	○	○	○	○	○	○	○	○

connected with others								
...made me feel good	◯	◯	◯	◯	◯	◯	◯	◯

How many times per week did you listen to music BEFORE COVID-19:

0,1,2,3,4,5,6,7, I prefer not to answer

For how long did you listen to music within the same day BEFORE COVID-19

0, 15 mins, 30 mins, 1 h, 2h,3,4,5, more than 5, I prefer not to answer

How many times per week do you listen to music DURING COVID-19:

0,1,2,3,4,5,6,7, I prefer not to answer

For how long do you listen to music within the same day DURING COVID-19

0, 15 mins, 30 mins, 1 h, 2h,3,4,5, more than 5, I prefer not to answer

If you have doubts or are experiencing any technical difficulties, please contact us at **steinhardt-marl-ripolleslab@nyu.edu**

Thank you for your time!

Is there anything else you would like to share regarding this survey?______

Write your email here if you want us to contact you to participate in other surveys in the future. Your email will be kept in an encrypted database of participants and there will be no link between your email and your responses to this survey. If you do not want to share this information, write 'No':__________

If you have doubts or are experiencing any technical difficulties, please contact us at **steinhardt-marl-ripolleslab@nyu.edu**
